# Nosocomial transmission of *Clostridium difficile* ribotype 027 in a Chinese hospital, 2012–2014, traced by whole genome sequencing

**DOI:** 10.1186/s12864-016-2708-0

**Published:** 2016-05-26

**Authors:** Hongbing Jia, Pengcheng Du, Hui Yang, Yuanyuan Zhang, Jing Wang, Wen Zhang, Guiling Han, Na Han, Zhiyuan Yao, Haiyin Wang, Jing Zhang, Zhen Wang, Qingming Ding, Yujun Qiang, Frédéric Barbut, George F. Gao, Yongtong Cao, Ying Cheng, Chen Chen

**Affiliations:** Department of clinical laboratory, China-Japan Friendship Hospital, No. 2 Yinghua Dongjie, Chaoyang, Beijing, 100029 China; Institute of Infectious Diseases, Beijing Ditan Hospital, Capital Medical University, and Beijing Key Laboratory of Emerging infectious Diseases, No. 8 Jingshundongjie, Beijing, 100015 China; State Key Laboratory for Infectious Disease Prevention and Control, and National Institute for Communicable Disease Control and Prevention, Chinese Center for Disease Control and Prevention, Beijing, 102206 China; Key Laboratory of Surveillance and Early-warning on Infectious Disease, Division of Infectious Disease, Chinese Center for Disease Control and Prevention, 155 Changbai Road, Changping, Beijing, 102206 China; National Reference Laboratory for Clostridium difficile, Faculté de Médecine Pierre et Marie Curie and Hôpital Saint-Antoine, Assistance Publique-Hôpitaux de Paris, Paris, 75012 France; Collaborative Innovation Center for Diagnosis and Treatment of Infectious Diseases, Hangzhou, 310003 China; The Institute of Microbiology, Chinese Academy of Sciences, Beijing, 100101 China

**Keywords:** *Clostridium difficile*, NAP1/BI/027, Outbreak, Nosocomial infection, Whole genome sequencing

## Abstract

**Background:**

The rapid spread of *Clostridium difficile* NAP1/BI/027 (*C. difficile* 027) has become one of the leading threats of healthcare-associated infections worldwide. However, *C. difficile* 027 infections have been rarely reported in Asia, particularly in China.

**Results:**

In this study, we identified a rare *C. difficile* bloodstream infection (BSI) from three isolates of a patient during repeated hospital admission. This finding triggered a retrospective epidemiological study to scan all cases and strains emerged from this ward during the past three years. Using medical personnel interviews, medical record reviews and the genomic epidemiology, two outbreaks in 2012 and 2013–2014 were identified. Through using whole genome sequencing, we succeeded to trace the origin of the BSI strain. Surprisingly, we found the genome sequences were similar to *C. difficile* 027 strain R20291, indicating the occurrence of a rare *C. difficile* 027 strain in China. Integrated epidemiological investigation and whole genome sequencing of all strains, we constructed a nosocomial transmission map of these two *C. difficile* 027 outbreaks and traced the origin of the infection.

**Conclusions:**

By genome sequencing, spatio-temporal analysis and field epidemiology investigation, we can estimate their complex transform network and reveal the possible modes of transmission in this ward. Based on their genetic diversity, we can assume that the toilets, bathroom, and janitor’s equipment room may be contaminated area, which may be suggested to improve infection control measures in the following health care.

**Electronic supplementary material:**

The online version of this article (doi:10.1186/s12864-016-2708-0) contains supplementary material, which is available to authorized users.

## Background

*Clostridium difficile* is an anaerobic, gram-positive, spore-forming bacterium responsible for infections ranging from mild diarrhea to pseudomembranous colitis, primarily in elderly patients exposed to antibiotics. Recently, this bacterium has become one of the most frequent microorganisms responsible for healthcare-associated infections in the United States [1]. During 2003, the first outbreak of a strain with “hyperproducer ” of toxin A and B, referred to as the North American Pulsed-field type one (NAP1), restriction-endonuclease analysis type BI, or PCR ribotype 027 (NAP1/BI/027), was reported in North America [2–5]. Since then, cases have been reported worldwide. Nevertheless, large outbreaks of NAP1/BI/027 are less reported in Asia and Latin America compared to North America and Europe [6, 7]. However, cases of NAP1/BI/027 infection have been already described in Hong Kong and Guangzhou, China [8, 9]. NAP1/BI/027 is characterized by an *in vitro* overproduction of toxins A and B as well as a binary toxin production, and an 18 bp deletion in *tcdC* gene [10]. Various epidemiological differences are noted in Asia; for example, the most common circulating ribotypes are 014, 017, and 018 [6]. Moreover, the toxin A-negative, B-positive (A^−^B^+^) strain 017 has received wide attention [6, 11]. To the best of our knowledge, no report of *C. difficile* infection (CDI) outbreaks in China has been published, and even few of case reported so far in the English literature.

In the present study, we report the first NAP1/BI/027 outbreak in a hospital in mainland China, traced by a rare case of bloodstream infection (BSI) using whole genome. This process of identifying outbreak was dramatic by a comprehensive retrospective investigation using temporal and spatial analysis of the cases and strain characterization including PCR-ribotyping, multi-locus sequence typing (MLST) and whole genome sequencing (WGS). The identification of the outbreak immediately provides four key implications: 1) the threat of CDI in China is more serious than that previously believed because of the under diagnosis of CDI in hospitals, and more cases were ignored; 2) the epidemiology of CDI should be carefully investigated to check whether there have been more cases of NAP1/BI/027 infection [11]; and 3) the infection control strategy should be strengthened. 4) WGS method may be helpful for investigating outbreaks and tracking strain transmission [12].

## Methods

### Case definition and CDI diagnosis

This study was conducted in the department of Traditional Chinese Medicine respiratory, a hospital of China from March 2012 to April 2014. A CDI case-patient was defined as a patient with 3 or more unformed stools in 24 or fewer consecutive hours and a stool test resulted positive for *C. difficile* toxin [13]. Stool samples were tested for toxin by enzyme immunoassay (*C. difficile* toxin A/B, Techlab, Blacksburg, VA, USA), and were anaerobically cultured on cycloserine-cefoxitin-fructose agar. Suspected colonies were identified by agglutination with *C. difficile* latex reagent for the somatic antigen (Oxoid Ltd, UK).

### Retrospectively epidemic investigation

A comprehensive retrospective epidemiological investigation was conducted. First, clinical information from medical records, including hospital stays, admitting diagnoses, antibiotic use, and occurrence of diarrhea were collected. Using these retrospective case searches, in total, 22 isolates were collected. Among 75 patients with diarrhea, 20 were diagnosed as CDI [13], including one patient with a *C. difficile* bloodstream infection. Symptom onset occurred after 48 h of hospitalization in 18 patients while 2 patients presented with diarrhea when admission. Second, the architectural structure of the ward and room arrangement were collected by field epidemiology investigation; at the same time, clinicians, nurses, and nurse-assistant were questioned about their behaviors during healthcare, paying particular attention to the overlap of hospital stays, use of the toilet and bathroom, and cleaning procedures.

### Validation and typing the strains in two outbreaks

In house-PCR was used to detect *tcdA* and *tcdB,* using the primer sets according to Lemee’s study [14], whereas MLST was used to allele-typing seven housekeeping genes, including *adk*, *atpA*, *dxr*, *glyA*, *recA*, *sodA* and *tpi* [15]. The isolates were characterized by a capillary gel electrophoresis-based ribotyping method using the NAP1/BI/027 strain UK1 as the reference [16].

### Genetic diversity, whole genome sequencing and bioinformatics analysis

A 500 bp library was constructed and whole genome sequencing was performed using Illumina Hiseq 2000 platform (Illumina, Inc., San Diego, CA, USA) to obtain a 100 bp paired-end read, with 1,463,765,173 bps in average for each sample (Accession number: SRP051490, Additional file 1: Table S2) [17]. The multi-alignment sequences from read data and references (ST1/027: R20291, accession number: NC_013316; ST37: M68, accession number: NC_017175) were obtained by the method that combines alignments from mappings to multiple reference sequences via REALPHY [18, 19], and then the phylogenetic tree was reconstructed via Bayesian evolutionary analysis by BEAST [20]. The genomic single nucleotide polymorphisms (SNPs) between NAP1/BI/027 strains were detected by mapping the *C. difficile* NAP1/BI/027 reference genome R20291 using SOAP2 software [21]. The sites with quality of <60 were removed. These SNPs were finally used to reconstruct the phylogenetic tree via Bayesian evolutionary analysis by BEAST [20]. The mutation pattern of NAP1/BI/027 was clustered by a hierarchical cluster algorithm using Cluster/Treeview software [22].

## Results

### Identification the BSI case of *C. difficile* NAP1/BI/027 infection

The patient was admitted on August 12, 2013 for community-acquired pneumonia. He was treated by cefepime (2000 mg 2 times per day) and levofloxacin (500 mg 1 time per day) intravenously for 7 days. On August 19, he displayed diarrhea, which progressively worsened. On August 22, his symptoms included fever, cramping, and abdominal discomfort. His stool sample was tested positive for the *C. difficile* toxin and culture. We simultaneously obtained his *C. difficile* isolate from an anaerobic blood culture. He was successfully treated with oral metronidazole (400 mg 3 times per day) for 13 days and then sequentially intravenous metronidazole (500 mg 3 times per day) for 4 days, and he was discharged on September 16. He was re-admitted three days later with persistent fever and diarrhea. Stool sample was positive again for toxin and culture but the blood culture was negative. He was treated with oral vancomycin (500 mg 3 times per day) for 12 days, and discharged on October 8. Clinical information and laboratory testing results of this patient (patient ID is P13) have been described in Additional file 1: Table S1.

### Retrospective study of clinical information of patients with CDI in the ward

**Fig. 1:**
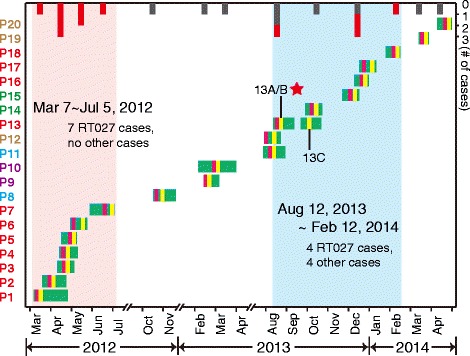
Occurrence of CDI in this ward from March 2012 to May 2014. The x-axis represents the time line and the y-axis represents the patient ID. The green bars represent the period of hospital stay of each patient, the purple and yellow lines represent the dates of bouts of diarrhea and C. difficile isolation, respectively. The isolate 13B from blood is marked by a red star (the same in other figures). The number of new cases in each month is displayed using vertical bars at the top, red for NAP1/BI/027 cases, and gray for remaining cases

This unusual BSI case due to *C. difficile* triggered a retrospective investigation of all CDI cases in the ward. In our record, nineteen additional patients with CDI were identified from March 2012 to April 2014. Their hospitalization periods are described in Fig. 1. All the patients were elderly (>65 y.) and had received antimicrobial treatments before diarrhea (Table 1). It is worth noting that CDI patients were more likely to be treated with third generation cephalosporins and fluoroquinolones than patients negative for CDI in the ward at the same time (Table 1). Two peaks of CDI occurred: one from March to July 2012 and the other from July 2013 to April 2014 suggesting two potential outbreaks (Fig. 1).

**Table 1:** Description of the patients with *C. difficile* infection

Characteristics	Value
No. of patients (%)	20 (100)
Males	14 (70.0)
Females	6 (30.0)
Age range (mean)	67–93 (80.9)
No. (%) of patients with nosocomial diarrhea	18 (90.0)
Length of hospital stay [range, days (mean)]	16–54 (28.6)
No. (%) of patients treated before diarrhea with the following	
Fluoroquinolones	13 (65.0)
4th generation cephalosporins	8 (40.0)
3rd generation cephalosporins	7 (35.0)
Carbapenems	5 (25.0)
Penicillins + β-lactamase inhibitor	2 (10.0)
Glycopeptides	1 (5.0)
Macrolides	1 (5.0)
Cephamycins	1 (5.0)

**Fig. 2:**
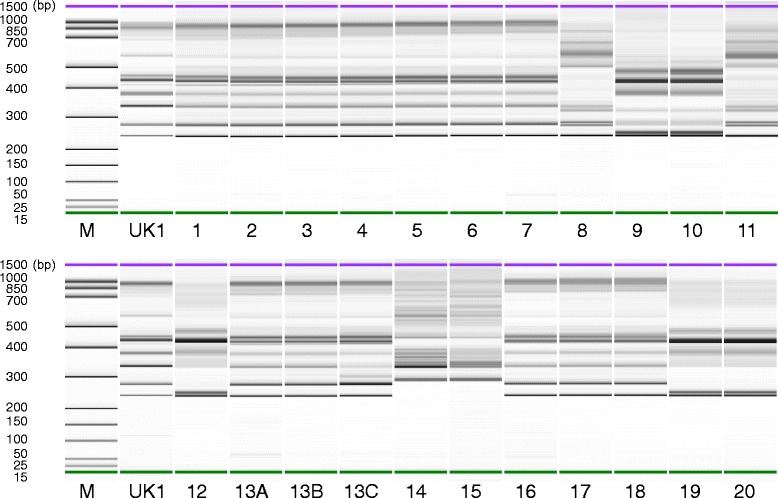
Capillary gel electrophoresis fingerprint of ribotyping of all *C. difficile* strains isolated from CDI patients in this study. Each lane represents as following: lane M, DNA marker (15 ~ 1500 bp); lane UK1, reference strain UK1 (NAP1/BI/RT027); other lanes, the 22 isolates from 20 CDI patients

All the positive strains of *C. difficile* isolated from stool samples were retained in our hospital. The clinical information and laboratory testing results have been presented in Additional file 1: Table S1. Seventeen strains were A + B+ and five were A − B+. MLST characterized these isolates into five sequence types (STs): ST1, ST2, ST8, ST37, and ST81. Eleven patients were infected by ST1 strain (A + B+, 11/20, 55 %) (Additional file 1: Table S1). Only two patients, P9 and P12 infected by ST37 and ST81, respectively, already presented CDI-caused diarrhea by the time of admission, which may be community-acquired. Thus, all ST1 infections were acquired within the hospital. Furthermore, all the ST1 strains were confirmed as ribotype 027 by capillary gel electrophoresis fingerprint of ribotyping (Fig. 2).

### Genetic population analysis in outbreaks

**Fig. 3:**
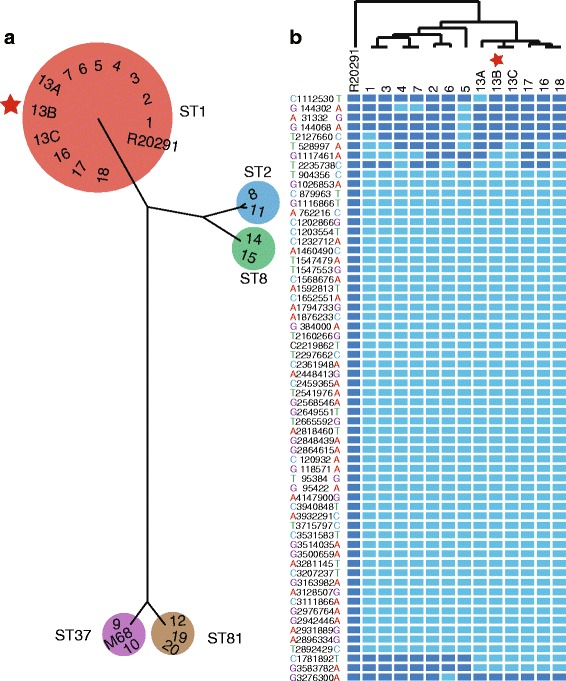
**a** Phylogenetic tree of 22 isolates from this study and two reference strains R20291 (ST1) and M68 (ST37) reconstructed by BEAST based on the multi-mapping results of REALPHY. The strain ID is coordinate to patient ID. **b** SNP pattern and relationship of NAP1/BI/027 isolates. Thirteen isolates from 11 cases (13A, 13B, and 13C were all from patient 13) are clustered using NAP1/BI/027 strain R20291 as the outgroup. The light blue squares represent mutations compared to R20291

All the isolated *C. difficile* strains were cultured and sequenced (Additional file 1: Table S2). After mapping all reads to the reference genomes of R20291 (NAP1/BI/027, ST1) and M68 (ST37), the multi-mapping results were combined and 3,404,190 bps aligned sequence was obtained from each genome, and then a Bayesian tree was reconstructed (Fig. 3a). The results revealed that all isolates were clearly characterized into five groups, as suggested by MLST (Additional file 1: Table S1, Fig. 3a), and the majority isolates from the outbreaks belonged to ST1. Moreover, more than thousands of SNPs were detected between ST1 and other types (Additional file 1: Table S3); therefore, ST2, ST8, ST81 and ST37 presented large genome diversity compared with published ST1 strains. These results confirmed that the strains belonging to different STs are not genetically related as which has been demonstrated before [23]. We also compared the SNPs within the same STs. Interestingly, when we scanned the genomes of ST1, most of them were close and tightly clustered together (Fig. 3a, Additional file 1: Table S4). We discovered only one SNP difference between isolates 4 and 7, illustrating their close relationship. These results suggest that ST1 isolates might come from the same origin and were from patient to patient transmissions. In addition to ST1, isolates within the other STs also clustered together. For example, two isolates belonging to ST37, presented a genetic distance with seven SNPs, and three isolates of ST81 presented one or two SNPs. These results suggest that isolates from the same ST may also come from the same origin. These results are in accordance with a former study in which 0–2 SNVs were identified between transmitted isolates obtained less than 124 days apart [24].

**Fig. 4:**
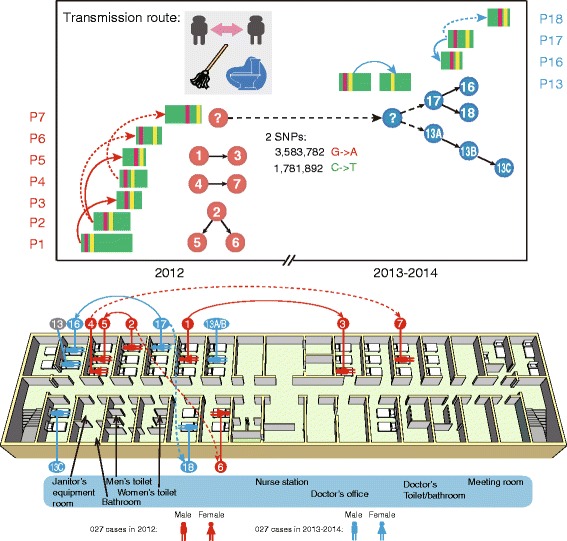
The temporal and spatial transmission map with three potential risks for *C. difficile* NAP1/BI/027 spreading. The transmission connections in two consecutive years were marked using blue and red lines, respectively. The full lines represent the potential transfer route with both genomics and epidemic evidence, and dashed lines connected the patients among whom only have potential genetic links. ”?” represented the unknown nodes during the transmission and evolution. The architectural structure, room arrangement, and distribution of NAP1/BI/027 cases in this ward were displayed at the bottom. Patients during the two outbreaks are distinguished by different colors of background: red for those in 2012 and blue for those in 2013 to 2014

By combining the clinical and laboratory information, we confirmed two outbreaks of NAP1/BI/027 had occurred, one from March to July 2012 and the other from December 2013 to February 2014 (Figs. 1 and 4).

### Whole genome analysis of NAP1/BI/027 isolates

After mapping the reads of NAP1/BI/027 (ST1) isolates to the reference genome R20291, a total of 62 SNPs were identified in our NAP1/BI/027 strains, including T1476G and G1514A located in *rpoB* gene associated with fluoroquinolone-resistance (Fig. 3b, Additional file 1: Table S4). All sequences from P13 were tightly clustered together, suggesting that these occurrences of CDI (once for BSI and twice for diarrhea) were related as we assumed (Fig. 2). The route of genetic transfer is clearly from 13A to 13B then to 13C, indicated by the genetic pattern with one SNP for each strain, although strain 13A presented an abnormal mutation. The results of WGS analysis confirmed our subsection that strain 13B causing BSI might have originated from the gut (Fig. 3b). The isolate 13C, responsible for CDI recurrence, was nearest to 13B from his blood. However, this hypothesis ignores the presence of within host diversity. The other possible alternation is that 13A, 13B and 13C may have existed simultaneously in the gut at both time points, and 13B caused BSI at the initial time. Since we cannot obtain multiple colony picks in this retrospective study, we empirically estimate that the gut infection lead to secondary BSI and gut persistence explains the relapse.

### Construction of the transmission map of NAP1/BI/027 for this outbreak

To reconstruct a putative transmission map, the clinical epidemiologic data were combined with the genetic data from results of WGS (Fig. 4). First, the temporal and spatial transmission links were constructed. The patients with overlapping time spent in bed were considered as the potential transmission connections (Fig. 4). Second, by conducting the field epidemiological investigation and consulting with heath care workers and patients, we assumed that the toilet and cleaning procedures were potential at high risk of transmission. Indeed, only one commode in the toilet and one mop were available in the ward, increasing the possible risk of the spore transmission among the patients. Last, we constructed the potential connections between the patients by genetic relationship among these strains. By combining all the data, particularly the genetic relationships between isolates and possible contacts between patients, we reconstructed the transmission routes of all NAP1/BI/027 cases to explain the outbreaks. Furthermore, our study indicated a trend between decreasing number of CDI and increasing distance to the janitor’s equipment room and toilet (Fig. 4).

Genome sequences provide the detailed molecular evidence to connect part of the transmission routes. First, phylogenetic analysis showed all NAP1/BI/027 isolates in this ward belonged to the same clone which existed in the ward in three years and caused two outbreaks, and we provided clear clues for explaining these outbreaks. Second, the isolates in these potential transfer routes shared most of the 62 SNPs, resulting in the direct connection among them (Fig. 4). For example, P3 only contains one SNP difference with P1, and this result indicates that these two patients may be infected by one strain or the pathogen may transfer from one to another. Similar results appeared in P7, P4 and P2, P5 and P6. Finally, WGS data also validate that the strains in two outbreaks in consecutive years were only different by 2 stable SNPs, indicating the infected strain may exist in the hospital and transfer again in the following year (Fig. 4). Based on the sequence diversity of our data set, the estimated time to the most recent common ancestor of the *C. difficile* strain was December 2011 (Additional file 1: Figure S2).

The potential connection of isolates according to time, space distribution, and molecular clues were integrated to define the full map of transmission routes (Fig. 4).

## Discussion

To our knowledge this is the first study investigating outbreaks of healthcare-associated *C. difficile* NAP1/BI/027 strain infection in China and reconstructing the transmission map by combining retrospective epidemiological analysis with whole-genome sequencing. The outbreak caused by this strain has not been reported in China, especially via bloodstream infection, which has high mortality. We provide evidence that WGS method could be used to tracing the source of infection and predicting the transmission routes.

*C. difficile* was the most commonly reported pathogen, accounting for 12.1 % of healthcare-associated infections [25]. The increased severity of CDI is primarily attributed to the emergence and rapid spread of the fluoroquinolone-resistant NAP1/BI/027 strain, which remains the most common clone worldwide [26]. Since the first large outbreak described in North America in 2003, the NAP1/BI/027 has become endemic in many hospital from North America, Europe, and more recently in Australia [2, 4, 27]. While, for a long time, lacking of the reports of CDI caused by the strain, makes us confusing about the states of 027 in China. Three major reasons may have contributed to this situation: 1) the major diagnostic method for CDI available in Chinese clinical laboratories is EIA for toxins A/B, and very few hospitals can perform *C. difficile* culture, resulting in many undetected CDI cases; 2) the lack of anaerobic laboratory equipment and limited CDI knowledge by healthcare staff also contributes to the poor isolation compliance to infection control measures and reporting under diagnosis of CDI in China; and 3) there is no reference center for typing *C. difficile* strains in China and identification of the different PCR ribotypes requires the collection of reference strains. Therefore, distribution of *C. difficile* circulating clones including the NAP/BI/027 is not known in China. PCR based sequencing method can detect the pathogen without reference strain by comparing with the public database. Thus, it becomes one of replacement to other method in lots of bacteria. However, not like other hospital acquired infection (HAI) bacteria, such as *Mycobacterium tuberculosis* and *Klebsiella pneumonia* [28], *C. difficile* is characterized by large genome diversity and instability increasing the difficulties to select proper primers for detection. This characteristic will increase the difficulty to extend the existed molecular typing methods used in US or UK to develop country, and easy to cause clinic false-negative results. So, the fact may be that: NAP1/BI/027 has existed in China before, yet it could not be identified immediately. Whole-genome sequencing can easily distinguish minor differences, providing valuable information for molecular typing, and pathogen or transmission route tracing [12, 29]. Thus, the genome complication would boost the application of WGS in *C. difficile* research. With the help of WGS, our research will enrich HAI pathogen database, and HAI outbreak tracing clues. Also, our research revealed the potential NAP/BI/027 threat for China will improve the completion of *C. difficile* NAP/BI/027 global epidemiology distribution [6, 30, 31].

In this study, we investigated CDI outbreaks using medical personnel interview, medical record review and WGS for strain typing. From the spatial analysis of the cases, we assumed that CDI patients are mainly concentrated near to the toilets, bathroom, and janitor’s equipment room (Fig. 4), which may be suggested to improve infection control measures in the following health care. However, we failed to obtain the isolates in environment. We also conducted special consultation with nurse-assistant and determined that the chlorine-containing disinfectant concentration (500 ppm available chlorine) used in the ward was lower than the concentration recommended for CDI infection control (with at least 1,000 ppm available chlorine). The epidemiological investigation revealed the absence of a dedicated area for CDI patient isolation, which was probably the most important contributor to the outbreak. P13, the first patient identified during the 2013–2014 outbreak, was placed in different hospital beds in the days following the emergence of diarrhea. In addition, this patient was neither isolated in a single room nor placed in contact precautions. To the best of our knowledge, person-to-person transmission was the main route of CDI transmission. However, the outbreak occurred during a long period spanning from 2012 to 2014, suggesting the existence of a potential environmental reservoir. For example, the hospitalized periods of patient four and seven had no overlap, and their rooms were quite apart away from each other (Fig. 4), showing that there was no obvious epidemiological connection by conventional methods. However, the WGS analysis revealed that there was only one SNP difference between isolates 4 and 7 from these two patients (Fig. 3b), which provides an important epidemiology clue and help to form epidemiology hypothesis. So, the method of WGS shows obvious potential superior to routine typing methods in epidemiology tracing. For example, we may confirm the transfer route of P13 and the clear route of P16, P17, and P18, which occurred during in the 2013–2014 outbreak. Furthermore, isolated strains from most cases in the outbreaks were sequenced belonging to ST1, and there were also several strains belonging to other STs with sufficient genetic diversity to ST1 in this study. These genetically distinct isolates may indicate diverse alternative sources or more complicated transmission modes, since *C. difficile* is an ancient organism with diverse strains present in humans, animals, and food. It is also noted that asymptomatic carriers might not be detected, which would have contributed to the transmission. The literature review revealed that approximately 50 % of the molecular resulting clusters can be confirmed by contact tracing documenting transmission links, particularly for a longitudinal time, space, and population span, which are difficult to establish using classic epidemiological links [32, 33]. Therefore, the molecular epidemiology based on WGS should be recommended for HAI investigations, in addition to conventional field epidemiology investigations.

Since we never found NAP1/BI/027 strain in the hospital before, this study is not carried out under a standard surveillance in the hospital, especially lack of pathogen information from environmental monitoring. All clinical trials were drawn back to collect after we identified and validated the strain, the complete transfer route of all strains in hospital is still not very clear. However, to our knowledge, this is the first case to identify *C. difficile* NAP/BI/027 outbreak by WGS method, which can be extended to other bacteria.

One of the most interesting features of the epidemic NAP1/BI/027 is high level of antibiotic resistance [30]. In the present study, we found the outbreak strain in China is consistent but different to that reported in other countries (Additional file 1: Table S4). For example, the *rpoB* gene presents the special mutations in T1476G and G1514A compared with R20291, in addition to the previously fluoroquinolone-resistance site reported [30]. In our cases, most patients were from the local community and were mainly admitted for chronic obstructive pulmonary disease and chronic bronchitis. The misuse of antibiotics, including the overuse and inadequate use of antibiotics, actually increased the antibiotic pressure and risk for CDI. In China, before 2012, patients suffering these diseases were usually prescribed antibiotics, particularly cephalosporins and fluoroquinolones. Additional file 1: Figure S1 shows that CDI patients were more likely to be treated with third generation cephalosporins and fluoroquinolones than patients negative for CDI in the ward at the same time. The results are in accordance with the previous publications [34]. Therefore, these observations highlight at least two noteworthy points: 1) primary medical clinicians should be aware of the potential CDI threat before the use of cephalosporins and fluoroquinolones to treat chronic obstructive pulmonary disease and chronic bronchitis of the elderly population and 2) the related family as well as healthcare workers caring for these elderly patients may also have potential *C. difficile* exposure risk, which should be particularly addressed. As we mentioned above, our cases emerged after the strictest antibiotic regulation has been implemented by the Chinese government in 2012. So, the regulations should be further enforced [35]. In addition, the existence and outbreak of *C. difficile* in our research may underscore the importance of monitoring antibiotic proper usage and taking preventive action to reduce transmission of such multidrug-resistant organisms in the hospital setting, thereby preventing nosocomial outbreaks. Thus, we propose that *C. difficile* surveillance must be conducted in health cares in China, Asia and other developing country, as an early warning of antibiotic resistance, allowing time for related detailed antibiotic interventions to be designed and implemented.

## Conclusions

By genome sequencing, spatio-temporal analysis and field epidemiology investigation of the *C. difficile* NAP1/BI/027 outbreaks in 2012 to 2014 in this ward, we can estimate their complex transform network, revealed the possible modes of transmission. Based on their genetic diversity, we can assume that the toilets, bathroom, and janitor’s equipment room may be contaminated area, which may be suggested to improve infection control measures in the following health care.

## Additional file

Additional file 1: Supplementary figures and tables. Figure S1. Comparison of Antibiotics Use Density (AUD) from *C. difficile* NAP1/BI/027 patients with that from all patients in this ward. Figure S2. The phylogenetic relationship and divergence time of *C. difficile* NAP1/BI/027 isolates calculated by BEAST. Table S1. Epidemiology of CDI patients and molecular typing of *C. difficile* isolates. Table S2. Genome sequencing status of all isolates. Table S3. The number of SNPs between different STs. Table S4. SNPs of *C. difficile* NAP1/BI/027 isolates in this study compared to R20291. (DOCX 122 kb)

## Abbreviations

BSI: bloodstream infection
CDI: *Clostridium difficile* infection
HAI: hospital acquired infection
MLST: multi-locus sequence typing
NAP1: the North American Pulsed-field type one
SNP: single nucleotide polymorphism
ST: sequence type
WGS: whole genome sequencing
