# Supplementary material for: Nosocomial transmission of Clostridium difficile ribotype 027 in a Chinese hospital, 2012–2014, traced by whole genome sequencing
Source: BMC Genomics. 2016 May 26;17:405. doi: 10.1186/s12864-016-2708-0 (PMC4942892; doi:10.1186/s12864-016-2708-0)
Supplement: Additional file 1: — Supplementary figures and tables. Figure S1. Comparison of Antibiotics Use Density (AUD) from C. difficile NAP1/BI/027 patients with that from all patients in this ward. Figure S2. The phylogenetic relationship and divergence time of C. difficile NAP1/BI/027 isolates calculated by BEAST. Table S1. Epidemiology of CDI patients and molecular typing of C. difficile isolates. Table S2. Genome sequencing status of all isolates. Table S3. The number of SNPs between different STs. Table S4. SNPs of C. difficile NAP1/BI/027 isolates in this study compared to R20291. (DOCX 122 kb) [file 12864_2016_2708_MOESM1_ESM.docx]

**Supplementary materials**

**Figure S1.** Comparison of Antibiotics Use Density (AUD) from *C. difficile* NAP1/BI/027 patients with that from all patients in this ward. (AUD= DDD/100 bed-days; DDD: Defined Daily Dose)


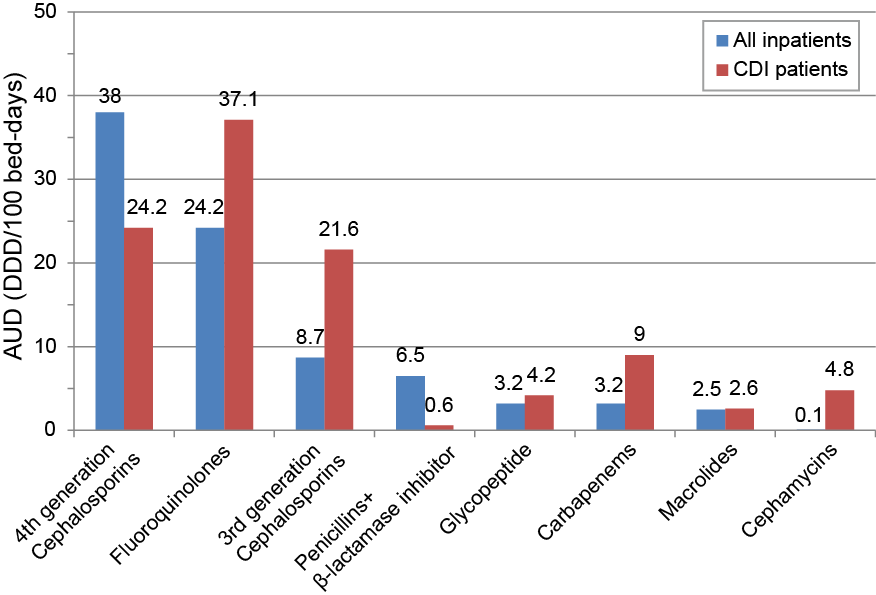


**Figure S2.** The phylogenetic relationship and divergence time of *C. difficile* NAP1/BI/027 isolates calculated by BEAST. The divergence time were displayed aside the nodes (year/month), and the axis at the bottom represents the timeline. The isolate 13B from blood were marked by a red star.


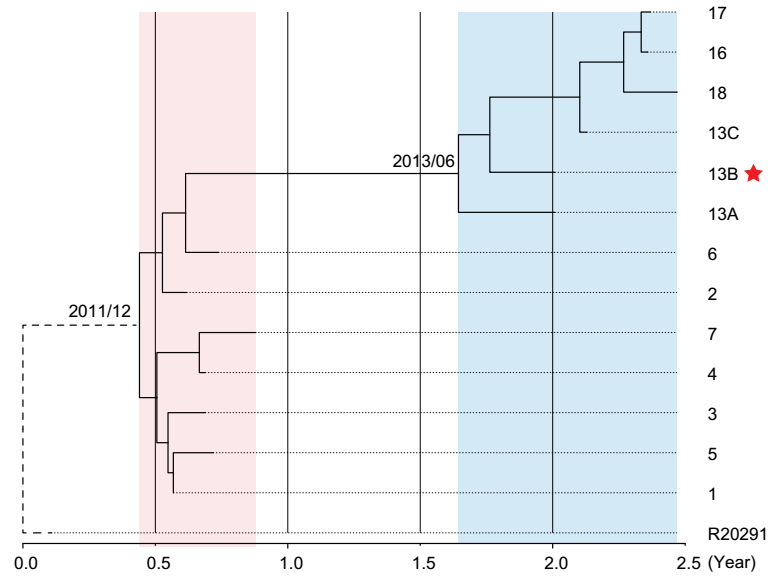


**Table S1.** Epidemiology of CDI patients and molecular typing of *C. difficile* isolates.

| **Patient ID^&^** | **Strain ID** | **Strain name** | **Toxino-typing** | **STs** | **Bed No.** | **Diarrhea before admission** | **Admitting diagnosis** | **Antibiotics used before nosocomial diarrhea** | **Date of Antibiotics used** | **Date of diarrhea** | **Sampling**  **date** | **Hospital stay** |
| --- | --- | --- | --- | --- | --- | --- | --- | --- | --- | --- | --- | --- |
| P1 | 1 | 1120 | A+B+ | ST1 | 17 | N | Thoracic Neoplasms | ZOX(4d) | 3/7/12 | 3/11/12 | 3/12/12 | 3/5 /12~4/24/12 |
| P2 | 2 | 1124 | A+B+ | ST1 | 24 | N | COPD | ZOX(18d),LVX(18d) | 3/13/12 | 4/1/12 | 4/2/12 | 3/13/12 ~4/19/12 |
| P3 | 3 | 1128 | A+B+ | ST1 | 10 | N | Dyspnea | ZOX(14d), MFX(1d) | 4/6/12 | 4/20/12 | 4/25/12 | 4/6 /12~5/6/12 |
| P4 | 4 | 1129 | A+B+ | ST1 | 25 | N | Lung infection | IPM(1d), SCF(4d) | 4/13/12 | 4/17/12 | 4/26/12 | 4/13/12 ~5/10/12 |
| P5 | 5 | 1131 | A+B+ | ST1 | 26 | N | Lung infection | MFX(7d) | 4/26/12 | 5/3/12 | 5/7/12 | 4/25/12 ~5/9/12 |
| P6 | 6 | 1132 | A+B+ | ST1 | 34 | N | Lung infection | ZOX(5d), LVX(5d) | 5/1/12 | 5/6/12 | 5/15/12 | 5/1/12 ~5/21/12 |
| P7 | 7 | 1136 | A+B+ | ST1 | 5 | N | Lung infection | FEP(24d), SCF(11d) | 6/3/12 | 6/23/12 | 7/5/12 | 5/30/12 ~7/5/12 |
| P8 | 8 | 1141 | A+B+ | ST2 | 12 | N | Lung infection | SCF(1d) | 10/20/12 | 10/21/12 | 10/29/12 | 10/19/12 ~11/20/12 |
| P9 | 9 | 1145 | A-B+ | ST37 | 13 | Y | Lung infection | P/ S(on the same day) | 2/18/13 | 2/18/13 | 2/19/13 | 2/18/13 ~3/6/13 |
| P10 | 10 | 1147 | A-B+ | ST37 | 25 | N | Pleural effusion | LVX(25d) | 2/7/13 | 3/4/13 | 3/5/13 | 2/7/13 ~3/31/13 |
| P11 | 11 | 1151 | A+B+ | ST2 | 15 | N | Lung infection | P/ S(1d),IPM(6d),MFX(9d),FEP(9d) | 7/29/13 | 8/7/13 | 8/7/13 | 7/29 /13~8/30/13 |
| P12 | 12 | 1156 | A-B+ | ST81 | 25 | Y | COPD | No record | No record | 8/1/13 | 8/9/13 | 8/1/13 ~8/20/13 |
| P13 | 13A, 13B* | 1157, 1158* | A+B+ | ST1 | 14/28# | N | Lung infection | FEP(8d),AZM(8d),IIPM(8d),LVX(8d) | 8/12/13 | 8/20/13 | 8/22/13 | 8/12/13 ~9/16/13 |
|  | 13C | 1164 | A+B+ | ST1 | 32 | Y | Fever, Diarrhea |  | - | - | 10/8/13 | 9/19/13 ~10/23/13 |
| P14 | 14 | 1161 | A+B+ | ST8 | 3 | N | Lung infection | MFX(10d),SCF(10d) | 9/29/13 | 10/9/13 | 10/14/13 | 9/29/13 ~10/25/13 |
| P15 | 15 | 1167 | A+B+ | ST8 | 1 | N | Lung infection | No record | No record | 12/9/13 | 12/12/13 | 11/29/13 ~12/19/13 |
| P16 | 16 | 1169 | A+B+ | ST1 | 29 | N | Lung infection | FEP(9d),MFX(4d) | 12/13/13 | 12/24/13 | 12/26/13 | 12/12/13 ~12/31/13 |
| P17 | 17 | 1172 | A+B+ | ST1 | 21 | N | Lung infection | MFX(2d) | 12/23/13 | 12/25/13 | 1/3/14 | 12/23/13 ~1/7/14 |
| P18 | 18 | 1173 | A+B+ | ST1 | +2 | N | Lung infection | FEP(12d),LVX(12d) | 1/22/14 | 2/3/14 | 2/6/14 | 1/22/14 ~2/12/14 |
| P19 | 19 | 1178 | A-B+ | ST81 | 16 | N | Lung infection | FEP(13d),IMP(13d),VAN(13d) | 3/7/14 | 3/20/14 | 3/20/14 | 3/7 /14~3/24/14 |
| P20 | 20 | 1184 | A-B+ | ST81 | 30 | N | Asthma | FOX(15d),LVX(15d) | 4/10/14 | 4/25/14 | 4/25/14 | 4/9 /14~4/30/14 |

& stool samples of 20 patients were tested positive for the C. difficile toxin A/B and culture all stool; *isolated from blood sample; #changing bed

M=male; F=female; Y=yes; N=no; ST= sequence type; COPD=chronic obstructive pulmonary diseases; ZOX=Ceftizoxime; LVX=Levofloxacin; MFX=Moxifloxacin; IPM=Imipenem; SCF=Cefoperazone/Sulbactam; FEP=Cefepime; P/S=Piperacillin/ Sulbactam; AZM=Azithromycin; VAN=Vancomycin; FOX=Cefoxitin

**Table S2.** Genome sequencing status of all isolates.

| **Patient ID** | **Strain ID** | **Strain name** | **Source** | **Accession number** | **Read** | | **Assembly** | | |
| --- | --- | --- | --- | --- | --- | --- | --- | --- | --- |
|  |  |  |  |  | **Clean base** | **Depth** | **Scaffold** | **Scaffold length** | **N50** |
| P1 | 1 | 1120 | Stool | SRR1735363 | 1,605,795,000 | 383.12 | 54 | 4,110,681 | 188,463 |
| P2 | 2 | 1124 | Stool | SRR1735364 | 1,350,401,200 | 322.19 | 42 | 4,098,520 | 219,716 |
| P3 | 3 | 1128 | Stool | SRR1735365 | 1,701,775,400 | 406.02 | 50 | 4,099,537 | 219,329 |
| P4 | 4 | 1129 | Stool | SRR1735366 | 1,661,972,800 | 396.53 | 42 | 4,109,646 | 219,386 |
| P5 | 5 | 1131 | Stool | SRR1735367 | 1,243,413,400 | 296.66 | 46 | 4,099,960 | 188,157 |
| P6 | 6 | 1132 | Stool | SRR1735368 | 1,218,529,200 | 290.73 | 45 | 4,117,043 | 220,187 |
| P7 | 7 | 1136 | Stool | SRR1735369 | 1,080,085,600 | 257.69 | 49 | 4,102,582 | 236,388 |
| P8 | 8 | 1141 | Stool | SRR1735370 | 1,741,883,200 | 415.59 | 33 | 4,033,930 | 371,145 |
| P9 | 9 | 1145 | Stool | SRR1735371 | 1,255,488,200 | 299.54 | 51 | 4,347,304 | 264,071 |
| P10 | 10 | 1147 | Stool | SRR1735372 | 1,730,976,200 | 412.99 | 47 | 4,373,517 | 244,762 |
| P11 | 11 | 1151 | Stool | SRR1735373 | 1,767,811,200 | 421.78 | 37 | 4,336,370 | 264,554 |
| P12 | 12 | 1156 | Stool | SRR1735374 | 1,431,224,800 | 341.47 | 46 | 4,283,954 | 222,893 |
| P13 | 13A | 1157 | Stool | SRR1735375 | 1,325,230,800 | 316.18 | 44 | 4,085,158 | 219,438 |
| P13 | 13B | 1158 | Blood | SRR1735376 | 1,584,679,200 | 378.08 | 43 | 4,117,159 | 230,818 |
| P13 | 13C | 1164 | Stool | SRR1735377 | 1,682,018,200 | 401.31 | 25 | 4,097,137 | 862,379 |
| P14 | 14 | 1161 | Stool | SRR1735378 | 1,253,506,800 | 299.07 | 47 | 4,081,242 | 219,456 |
| P15 | 15 | 1167 | Stool | SRR1735379 | 1,520,626,200 | 362.80 | 40 | 4,224,317 | 285,434 |
| P16 | 16 | 1169 | Stool | SRR1735380 | 1,405,547,200 | 335.35 | 56 | 4,095,723 | 142,685 |
| P17 | 17 | 1172 | Stool | SRR1735381 | 1,274,374,400 | 304.05 | 45 | 4,094,561 | 220,136 |
| P18 | 18 | 1173 | Stool | SRR1735382 | 1,464,093,800 | 349.31 | 43 | 4,097,813 | 196,681 |
| P19 | 19 | 1178 | Stool | SRR1735383 | 1,604,316,200 | 382.77 | 44 | 4,273,114 | 225,850 |
| P20 | 20 | 1184 | Stool | SRR1735384 | 1,299,084,800 | 309.95 | 43 | 4,266,893 | 230,205 |

**Table S3. The number of SNPs between different STs**

| **STs** | **Isolates** | **# of SNPs** | | | | | | | | | | |
| --- | --- | --- | --- | --- | --- | --- | --- | --- | --- | --- | --- | --- |
|  |  | **R20291** | **14** | **15** | **8** | **11** | **12** | **19** | **20** | **M68** | **9** | **10** |
| ST1 | R20291 | 0 | 20680 | 20668 | 21326 | 21185 | 42657 | 42657 | 42656 | 46334 | 46338 | 46341 |
| ST8 | 14 | 20680 | 0 | 94 | 9643 | 9357 | 39059 | 39059 | 39058 | 45348 | 45348 | 45351 |
| ST8 | 15 | 20668 | 94 | 0 | 9633 | 9343 | 39053 | 39053 | 39052 | 45334 | 45340 | 45343 |
| ST2 | 8 | 21326 | 9643 | 9633 | 0 | 1450 | 39739 | 39739 | 39738 | 44939 | 44945 | 44948 |
| ST2 | 11 | 21185 | 9357 | 9343 | 1450 | 0 | 39800 | 39800 | 39799 | 45023 | 45029 | 45032 |
| ST81 | 12 | 42657 | 39059 | 39053 | 39739 | 39800 | 0 | 2 | 1 | 6342 | 6349 | 6350 |
| ST81 | 19 | 42657 | 39059 | 39053 | 39739 | 39800 | 2 | 0 | 1 | 6342 | 6349 | 6350 |
| ST81 | 20 | 42656 | 39058 | 39052 | 39738 | 39799 | 1 | 1 | 0 | 6341 | 6348 | 6349 |
| ST37 | M68 | 46334 | 45348 | 45334 | 44939 | 45023 | 6342 | 6342 | 6341 | 0 | 43 | 44 |
| ST37 | 9 | 46338 | 45348 | 45340 | 44945 | 45029 | 6349 | 6349 | 6348 | 43 | 0 | 7 |
| ST37 | 10 | 46341 | 45351 | 45343 | 44948 | 45032 | 6350 | 6350 | 6349 | 44 | 7 | 0 |

**Table S4.** SNPs of *C. difficile* NAP1/BI/027 isolates in this study compared to R20291.

| **Position** | **Strain name of mutations** | **CDS** | **Distance to CDS*** | **Reference base** | **Reference residue** | **Query base** | **Query residue** | **Function** |
| --- | --- | --- | --- | --- | --- | --- | --- | --- |
| 3,276,300 | 1132 | CDR20291_2766 | -54 | G | - | A | - | 2-keto-3-deoxygluconate permease |
| 1,112,530 | 1157 | CDR20291_0899 | 893 | C | S | T | L | exonuclease subunit C |
| 31,332 | 1131 | CDR20291_0009 | -2598 | A | - | G | - | hypothetical protein |
| 144,068 | 1131 | CDR20291_0107 | +1331 | G | - | A | - | anaerobic ribonucleoside-triphosphate reductasactivating protein |
| 2,127,660 | 1120, 1131 | CDR20291_1810 | -1429 | T | - | C | - | hypothetical protein |
| 144,302 | 1129, 1136, 1131 | CDR20291_0107 | +1565 | G | - | A | - | anaerobic ribonucleoside-triphosphate reductasactivating protein |
| 528,997 | 1120, 1128, 1131, 1164, 1169, 1173 | CDR20291_0440 | +29 | T | - | A | - | hemagglutinin/adhesin |
| 1,117,461 | 1120, 1128, 1129, 1136, 1158, 1164 | CDR20291_0902 | -391 | G | - | A | - | peptidase T |
| 2,235,738 | 1129, 1132, 1157, 1158, 1164, 1173 | CDR20291_1913 | 246 | T | P | C | P | hypothetical protein |
| 1,781,892 | 1157, 1158, 1164, 1172, 1169, 1173 | CDR20291_1513 | 416 | C | A | T | V | sodium extrusion ABC transporter permease |
| 3,583,782 | 1157, 1158, 1164, 1172, 1169, 1173 | CDR20291_3004 | -12 | G | - | A | - | phage DNA-binding protein |
| 95,384 | All | CDR20291_0060 | 1476 | T | Q | G | Q | DNA-directed RNA polymerase subunit beta |
| 95,422 | All | CDR20291_0060 | 1514 | G | R | A | K | DNA-directed RNA polymerase subunit beta |
| 118,571 | All | CDR20291_0090 | 187 | G | D | A | N | ribosomal protein |
| 120,932 | All | CDR20291_0096 | 174 | C | D | A | E | DNA-directed RNA polymerase subunit alpha |
| 384,000 | All | CDR20291_0319 | 218 | G | W | A | * | hypothetical protein |
| 762,216 | All | CDR20291_0616 | +96 | A | - | C | - | nucleotide phosphodiesterase |
| 879,963 | All | CDR20291_0712 | 1663 | C | H | T | N | penicillin-binding protein |
| 904,356 | All | CDR20291_0735 | 31 | T | S | C | P | electron transfer flavoprotein subunit beta |
| 1,026,853 | All | CDR20291_0842 | 567 | G | A | A | A | 2-isopropylmalate synthase |
| 1,116,866 | All | CDR20291_0900 | +305 | G | - | T | - | modulator of ions transport |
| 1,202,866 | All | CDR20291_0985 | 1489 | C | L | G | V | penicillin-binding protein |
| 1,203,554 | All | CDR20291_0985 | 2177 | C | A | T | V | penicillin-binding protein |
| 1,232,712 | All | CDR20291_1013 | 197 | C | T | A | N | hypothetical protein |
| 1,460,490 | All | CDR20291_1231 | 719 | A | D | C | A | transporter |
| 1,547,479 | All | CDR20291_1308 | 463 | T | F | A | F | 5-nitroimidazole reductase |
| 1,547,553 | All | CDR20291_1308 | 389 | T | T | G | T | 5-nitroimidazole reductase |
| 1,568,676 | All | CDR20291_1323 | 412 | C | Q | A | K | ruberythrin |
| 1,592,813 | All | CDR20291_1347 | -67 | A | - | T | - | hypothetical protein |
| 1,652,551 | All | CDR20291_1396 | 241 | C | L | A | I | ATP phosphoribosyltransferase regulatorsubunit |
| 1,794,733 | All | CDR20291_1522 | 343 | A | T | G | A | two-component response regulator |
| 1,876,233 | All | CDR20291_1593 | 1169 | A | T | C | S | arsenical pump membrane protein |
| 2,160,266 | All | CDR20291_1848 | 922 | T | S | G | A | peptidase |
| 2,219,862 | All | CDR20291_1900 | 863 | C | C | T | Y | DNA mismatch repair protein |
| 2,297,662 | All | CDR20291_1968 | 329 | T | I | C | S | hypothetical protein |
| 2,361,948 | All | CDR20291_2018 | +54 | C | - | A | - | DeoR family transcriptional regulator |
| 2,448,413 | All | CDR20291_2088 | 164 | A | T | G | T | ATP-dependent RNA helicase |
| 2,459,365 | All | CDR20291_2096 | 605 | C | * | A | L | cyclomaltodextrinase |
| 2,541,976 | All | CDR20291_2164 | 533 | T | W | A | L | hypothetical protein |
| 2,568,546 | All | CDR20291_2183 | -246 | G | - | A | - | beta-lactamase inducer |
| 2,649,551 | All | CDR20291_2259 | 205 | G | * | T | K | quinolinate synthetase |
| 2,665,592 | All | CDR20291_2272 | 684 | T | L | G | L | signaling protein |
| 2,818,460 | All | CDR20291_2406 | -69 | A | - | T | - | threonine dehydratase catabolic |
| 2,848,439 | All | CDR20291_2429 | 207 | G | S | A | S | hypothetical protein |
| 2,864,615 | All | CDR20291_2442 | +140 | G | - | A | - | hypothetical protein |
| 2,892,429 | All | CDR20291_2467 | 1238 | T | F | C | C | ribosomal RNA small subunit methyltransferase |
| 2,896,334 | All | CDR20291_2471 | 311 | A | T | G | T | peptide deformylase 2 |
| 2,931,889 | All | CDR20291_2499 | 34 | A | M | G | L | hypothetical protein |
| 2,942,446 | All | CDR20291_2509 | -60 | G | - | A | - | hypothetical protein |
| 2,976,764 | All | CDR20291_2541 | 902 | G | Y | A | F | UDP-N-acetylmuramoylalanine--D-glutamate ligase |
| 3,111,866 | All | CDR20291_2643 | 981 | C | I | A | I | phosphoenolpyruvate-protein phosphotransferase |
| 3,128,507 | All | CDR20291_2657 | 1740 | A | N | G | N | capsular polysaccharide biosynthesis protein |
| 3,163,982 | All | CDR20291_2682 | 467 | G | F | A | F | S-layer precursor protein |
| 3,207,237 | All | CDR20291_2713 | 478 | C | H | T | Y | hypothetical protein |
| 3,281,145 | All | CDR20291_2769 | 2009 | A | Y | T | F | signaling protein |
| 3,500,659 | All | CDR20291_2948 | +417 | G | - | A | - | 6-phosphofructokinase |
| 3,514,035 | All | CDR20291_2957 | +115 | G | - | A | - | hypothetical protein |
| 3,531,583 | All | CDR20291_2969 | 1165 | C | N | T | N | PTS system transporter subunit IIABC |
| 3,715,797 | All | CDR20291_3112 | -322 | T | - | C | - | serine protease |
| 3,932,291 | All | CDR20291_3294 | 996 | A | L | C | L | hypothetical protein |
| 3,940,848 | All | CDR20291_3304 | 224 | C | L | T | H | F0F1 ATP synthase subunit beta |
| 4,147,900 | All | CDR20291_3497 | 1216 | A | S | G | P | hypothetical protein |

*: "-" and "+" represent the mutation located upstream or downstream the gene. The numbers represent the distance to the start.
